# Relationship between the miRNA Profiles and Oncogene Mutations in Non-Smoker Lung Cancer. Relevance for Lung Cancer Personalized Screenings and Treatments

**DOI:** 10.3390/jpm11030182

**Published:** 2021-03-05

**Authors:** Alberto Izzotti, Gabriela Coronel Vargas, Alessandra Pulliero, Simona Coco, Irene Vanni, Cristina Colarossi, Giuseppina Blanco, Antonella Agodi, Martina Barchitta, Andrea Maugeri, CT-ME-EN Cancer Registry Workers, Gea Oliveri Conti, Margherita Ferrante, Salvatore Sciacca

**Affiliations:** 1 Department of Experimental Medicine, University of Genoa, 16132 Genoa, Italy; 2 UOC Mutagenesis and Cancer Prevention, IRCCS Ospedale Policlinico San Martino, 16132 Genoa, Italy; 3 Department of Health Sciences, University of Genoa, 16132 Genoa, Italy; S3563836@unige.it (G.C.V.); alessandra.pulliero@unige.it (A.P.); 4 Lung Cancer Unit, IRCCS Ospedale Policlinico San Martino, 16132 Genoa, Italy; simona.coco@hsanmartino.it (S.C.); irene.vanni@sanmartino.it (I.V.); 5 Genetics of Rare Cancers, Department of Internal Medicine and Medical Specialties, University of Genoa, 16132 Genoa, Italy; 6 Department of Experimental Oncology, Mediterranean Institute of Oncology (IOM), 95029 Catania, Italy; cristina.colarossi@grupposamed.com (C.C.); giusi.blanco@grupposamed.com (G.B.); marfer@unict.it (M.F.); tsciacca42@gmail.com (S.S.); 7 Department of Medical and Surgical Sciences and Advanced Technologies “G.F. Ingrassia”, University of Catania, 95123 Catania, Italy; agodia@unict.it (A.A.); martina.barchitta@unict.it (M.B.); andrea.maugeri@unict.it (A.M.); olivericonti@unict.it (G.O.C.); 8 Catania, Messina, Enna Cancer Registry, Via S. Sofia 87, 95123 Catania, Italy; segreteria@registrotumoriintegrato.it

**Keywords:** nonsmokers lung cancer, miRNA, environmental risk factors, oncogenes, mutations

## Abstract

Oncogene mutations may be drivers of the carcinogenesis process. MicroRNA (miRNA) alterations may be adaptive or pathogenic and can have consequences only when mutation in the controlled oncogenes occurs. The aim of this research was to analyze the interplay between miRNA expression and oncogene mutation. A total of 2549 miRNAs were analyzed in cancer tissue—in surrounding normal lung tissue collected from 64 non-smoking patients and in blood plasma. Mutations in 92 hotspots of 22 oncogenes were tested in the lung cancer tissue. MicroRNA alterations were related to the mutations occurring in cancer patients. Conversely, the frequency of mutation occurrence was variable and spanned from the k-ras and p53 mutation detected in 30% of patients to 20% of patients in which no mutation was detected. The prediction of survival at a 3-year follow up did not occur for mutation analysis but was, conversely, well evident for miRNA analysis highlighting a pattern of miRNA distinguishing between survivors and death in patients 3 years before this clinical onset. A signature of six lung cancer specific miRNAs occurring both in the lungs and blood was identified. The obtained results provide evidence that the analysis of both miRNA and oncogene mutations was more informative than the oncogene mutation analysis currently performed in clinical practice.

## 1. Introduction

MicroRNAs (miRNAs) are non-coding RNA molecules that have different regulatory roles in cell differentiation, proliferation, and survival. miRNAs can inhibit complementary mRNA targets, regulating translation through RNA degradation. miRNAs were found to be deregulated in numerous diseases, including cancer, and are frequently altered owing to mutations or transcriptional changes of the enzymes that regulate miRNA biogenesis [1]. miRNAs are also involved in the epithelial–mesenchymal transition, cell growth, proliferation, migration, and invasion [2], as well as processes related to chemotherapy cell resistance. For example, miR-92a expression is increased in *PTEN* deletion cases [3], miR-244 is related to the apoptosis process enhancing the proliferative and migratory effects in non-small cell lung cancer (NSCLC) [4], and miR-200c influences the epithelial–mesenchymal transition in A549 cells [5].

Experimental findings have linked environmental exposure, carcinogenesis, and miRNA profiles. Neither epigenetic nor genetic alteration, when used alone, accurately predict the lung cancer risk in exposed subjects. Indeed, the adverse effects of mutations can be silenced by a functional microRNA machinery and the alteration of the miRNA machinery is devoid of remarkable consequences in absence of genotoxic damage. Early diagnosis of lung cancers using miR-33a-5p and miR-128-3p signatures have been proposed as they are linked to tumor suppression processes [6]. These findings address the identification of a cluster of miRNAs to be used as cancer early predictors considering the high heterogeneity of lung cancer patients.

However, the use of microRNA analysis alone for early lung cancer detection is still questionable. Indeed, despite a variety of miRNA being released extracellularly in the blood from growing cancers [7], the use of blood circulating miRNAs for cancer diagnosis is still a research matter and is not yet applicable to clinical and preventive practices. Several problems still hamper the on-field application of miRNA analysis as a tool for preventive medicine, including (a) the lack of correspondence between cancer and blood miRNAs; (b) the overwhelming effect of miRNA released from large mass organs on cancer miRNAs; (c) the poor reproducibility of miRNA cancer signatures among different studies; (d) the limited predictivity of miRNA analysis when used alone.

MicroRNAs are released not only from growing cancer but also, under physiological conditions, by organs, such as the skeletal muscle, liver, and kidneys (weighting kilos) [8]. The amount of these miRNAs overwhelms those released from the low-size growing cancer mass (weight of a few mg and size of a few mm), a problem increased by the fact that miRNAs are only partly tissue specific, as the same miRNA is expressed, although at different levels, in various body tissues.

The limited predictivity for the cancer occurrence of miRNAs when used alone is because miRNAs are quite unspecific with each one regulating hundreds of genes and, not only one but many, miRNAs play a pathogenic role in cancer progression. The main anti-cancer mechanism exerted by miRNA is the suppression of messenger RNAs produced by mutated oncogenes. This situation occurs in lung cancer for let-7 miRNA suppressing the expression of mutated k-ras oncogene [9] in breast cancer for miR-335 modulating the expression and the biological effects of the mutated BRCA1 oncogene [10].

Accordingly, whenever an oncogene is mutated, but its suppressing miRNA is still functioning, there is no push toward cancer progression, and thus the mutation is devoid of clinical predictivity. Similarly, whenever a microRNA is altered or downregulated, but its controlled genes is not mutated, again, there is no push toward cancer progression, and thus the microRNA alteration mutation is devoid of clinical predictivity. Giving this situation, it appears to be of high relevance to test, in parallel, both microRNA alterations and oncogene mutations to increase the clinical predictivity of these cancer biomarkers.

The study was focused on non or ex-smokers only. Indeed, cigarette smoke exerts a well-documented direct alteration of miRNA expression [11]. Accordingly, we decided to limit the effect of this confounding factor focusing on the relationship between miRNA and oncogenes as related to lung cancer only. This design was more difficult than recruiting current smokers; however, this was the only feasible approach to guarantee that the obtained results reflected cancer-related effects only and were not the results of the direct action of cigarette smoke on the miRNA machinery.

The aim of the herein reported research project is to use integrated mutations and microRNA expression as a new tool to perform lung cancer diagnosis and to identify high risk subjects with reference to lung cancer in non-smokers.

## 2. Materials and Methods

### 2.1. Patient Recruitment and Sampling

The enrollment of the patients was performed in the four biggest hospitals of Catania (University Hospital “G. Rodolico—San Marco”, “Garibaldi-Nesima” Hospital, “Cannizzaro” Hospital, and “Morgagni” Clinic), and in the “San Vincenzo” Hospital of Taormina of Messina province.

The protocol of this study was approved by the Ethics Committees (n. 11778 released on 17 March 2015. and 346/C.E. released on 28 May 2015) of the involved institutions and performed according to the Declaration of Helsinki.

We used the following inclusion criteria for the patient enrollment: age > 18 years, undergoing lung cancer-surgery, being non-smokers or former smokers for at least 5 years, survival within 3 years, the exclusion of other concurrent diseases, and having signed the written informed consent during the interview. No gender restriction was considered, and no restricted selection was performed regarding the morphology of the reported neoplastic lesions. From the same patient, both neoplastic and healthy tissue samples (lung tissue biopsy) were taken.

The lung tissue samples were sampled directly from the pathological anatomies of the hospitals involved in the project. Instead, the venous blood samples were collected directly from the thoracic surgery units of the hospitals involved. A total of 64 patients were enrolled, including 42 males (66%) and 22 females (34%), aged 69.0 ± 9.5 years, min 43 and max 84 years old.

Through the questionnaire, socio-demographic and lifestyle information, including smoking history, nutrition, home characteristics, and home location, were collected. From the 64 patients enrolled we sampled 64 blood samples, of which, 41 blood samples and 52 lung tissues were dedicated to miRNA profiling and oncogene mutation analysis by Ion-Torrent sequencing. For 35 patients, a 3 years follow up was performed to evaluate their clinical status. These 35 patients were referred to as ‘monitored patients’.

### 2.2. DNA Extraction

Genomic DNA (gDNA) was extracted from 25 mg of fresh frozen lung biopsy DNA using the DNeasy Blood & Tissue kit (Qiagen, Milan, Italy), as described by the manufacturer’s protocol. The purification of gDNA was automated on the QIAcube instrument (Qiagen, Milan, Italy). The gDNA quality and quantity were assessed with a NanoDrop 1000 spectrometer and with a Qubit 3.0 Fluorometer using a dsDNA HS Assay Kit (Thermo Fisher Scientific, Carlsbad, CA, USA).

### 2.3. Somatic Mutation Identification

The mutational status of 22 oncogenes (*KRAS*, *EGFR*, *BRAF*, *PIK3CA*, *AKT1*, *ERBB2*, *PTEN*, *NRAS*, *STK11*, *MAP2K1*, *ALK*, *DDR2*, *CTNNB1*, *MET*, *TP53*, *SMAD4*, *FBX7*, *FGFR3*, *NOTCH1*, *ERBB4*, *FGFR1*, and *FGFR2*) associated with lung cancer was analyzed by sequencing using the Colon and Lung Cancer Research Panel v.2 (Thermo Fisher Scientific, Carlsbad, CA, USA), which screens 92 amplicons in hotspots and target regions of these genes. For each sample, 15 ng of gDNA was amplified using the Ion AmpliSeq™ Library Kit 2.0 (Thermo Fisher Scientific, Carlsbad, CA, USA) according to the protocol for gDNA isolated from fresh frozen samples [12].

The quality control of the libraries was assessed by TapeStation 2200 using the High Sensitivity D1000 assay (Agilent Technologies, Santa Clara, CA, USA) and with a Qubit^®^ 2.0 Fluorometer using the dsDNA HS Assay Kit (Thermo Fisher Scientific, Carlsbad, CA, USA). Then, seven multiplexed libraries (100 pM) were amplified and enriched by OneTouch™ and the OneTouch™ ES, respectively using Ion PGM™ Hi-Q™ View OT2 Kit (Thermo Fisher Scientific, Carlsbad, CA, USA). Finally, the template was loaded onto a 316 v.2 chip and sequenced using the Ion PGM™ Hi-Q™ View Sequencing Kit on the Ion PGM™ platform (Thermo Fisher Scientific, Carlsbad, CA, USA). The sequencing data were analyzed using the Ion Torrent Software Suite with the plugin Torrent Variant Caller v.5.10.0.18 (Thermo Fisher Scientific, Carlsbad, CA, USA) applying somatic, high stringency parameters. We considered gene variants with a variant allele frequency up to 1%, if covered at least 1000×. All gene variants were annotated by Ion Reporter™ Software v. 5.10.

### 2.4. Total RNA Extraction 

The total RNA was extracted from lung biopsies and blood plasma using standardized protocols that combined phenol/guanidine-based lysis of samples and silica-membrane-based purification. 

Briefly, 3 mL of whole blood were collected in Ethylenediaminetetraacetic acid (EDTA) tubes and layered onto 3 mL Histopaque-1077 (Sigma-Aldrich Chemie Gmbh, Munich, Germany) through centrifugation at 400× *g* for 30 min. Plasma and lymphocytes were separately collected and stored at −20 °C at the Laboratory of Molecular Epidemiology (University of Catania) until analysis. Next, the total RNA from the plasma was extracted using the miRNeasy Serum/Plasma Kit (Qiagen, Milan, Italy), as described by the manufacturer’s protocol. 

With respect to lung biopsies, 30 mg of fresh starting material was first stabilized in 2.5 mL of RNAlater solution and stored at −20 °C at the Laboratory of Molecular Epidemiology (University of Catania) until analysis. Next, lung biopsies were disrupted using the TissueRuptor II for 20–40 s and homogenized in 700 µL QIAzol Lysis Reagent (Qiagen, Milan, Italy). The total RNA was purified from the homogenate using the miRNeasy Mini Kit (Qiagen, Milan, Italy), as described by the manufacturer’s protocol. The purification of RNA was automated on the QIAcube instrument (Qiagen, Milan, Italy). The quantification of RNA was assessed with a qubit 3.0 Fluorometer using the HS RNA Assay kit (Thermo Fisher Scientific, Carlsbad, CA, USA).

### 2.5. MiRNA Microarray Analysis

MiRNA profiling was performed by Agilent Platform using Human miRNA 8 × 60 K Microarray containing 2549 miRNAs (miRBase 21.0) (Agilent Technologies, Santa Clara, CA, USA). For each sample, 50 ng of the total RNA, including the miRNAs, was labeled, and hybridized according to the manufacturer’s instructions for miRNA Complete Labeling and the Hyb protocol (v. 3.1.1). The hybridized microarrays were acquired using the G2565CA scanner (Agilent Technologies) and the images were processed by Feature Extraction software v.9.5.3.1 (Agilent Technologies, Santa Clara, CA, USA). All raw data were loaded in the Gene Expression Omnibus (http://www.ncbi.nlm.nih.gov/geo/; GEO number accession requested, 8 March 2021). A tab-delimited text file was analyzed in the R v. 2.7.2 software environment http://www.r-project.org (accessed on 8 March 2021) using the limma package v.2.14.16 of Bioconductor http://www.bioconductor.org accessed on 8 March 2021.

Only spots with a signal minus background that were flagged as positive and significant were used in the following analysis as ‘detected’ spots. Probes with less than 50% of detected spots across all arrays, and arrays with several detected spots smaller than 50% of all spots on the array were removed. The background-corrected intensities of the replicated spots on each array were averaged. The data were then log2-transformed and normalized for between-array comparison using quintile normalization [13]. MicroRNAs with *p*-values < 0.05 were selected for further analysis. Given the explorative nature of this study, no correction for multiple testing was applied in the screening procedure aimed at selecting multiple sets of microRNAs for subsequent hierarchical clustering analyses. The agglomerative hierarchical clusters, used to detect similarity relationships in microRNA log2-transformed expressions, were computed using the Euclidean distance between single vectors and the Ward method [14].

### 2.6. Statistical Analysis

GeneSpring software (GeneSpring Multi-Omic Analysis version 14.9–Build 11939 by Agilent Technologies) was used for the analysis of miRNA expression from lung-tumoral (*T* = 38), lung-healthy (*S* = 12), and blood tissues (*n* = 41). 

All lung-tissue-miRNA raw data files from the Agilent Technologies Microarray Scanner System G2505C were imported to GeneSpring using miRNA analysis type, Technology 70156_v21_0, without baseline transformation. The blood miRNA chip raw data were import to GeneSpring with a custom technology as scanner analysis technology was not available. The protocol used was: Analysis type = Expression, Experiment type = Generic Single Color, Normalization algorithm = none, percentile target = 75, and baseline transformation = none. 

## 3. Results

### 3.1. Mutations in Oncogenes

**Figure 1:**
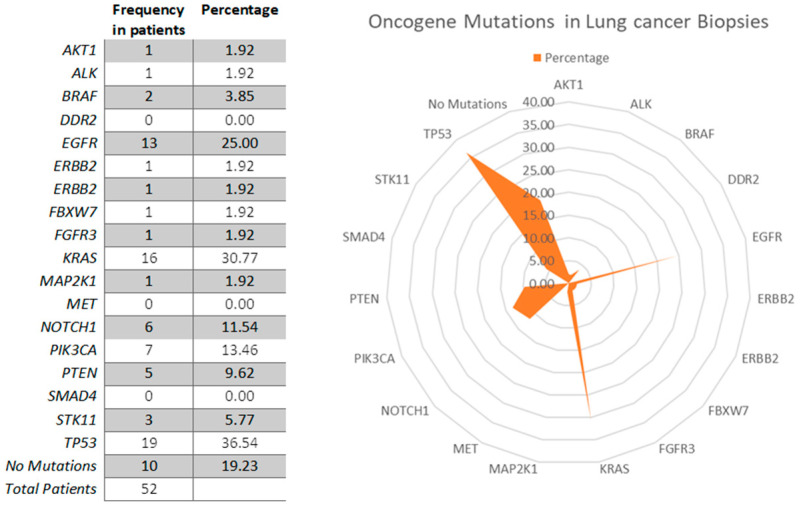
Radar chart showing the frequency of mutations in the lung biopsies of analyzed patients. *TP53* (36.54%), *KRAS* (30.77%), *EGFR* (25%), *PIK3CA* (13.46%), and *NOTCH1* (11.54%) were the most frequent mutations in the analyzed patients.

Despite the presence of lung cancer, no hotspot mutation was observed in 10 out of the 52 examined patients (19.2%). In 42 patients, mutations in oncogenes were observed with the following frequency ranking: *TP53* (36.54%), *KRAS* (30.77%), *EGFR* (25%), *PIK3CA* (13.46%), *ERBB2* (1.92%), *STK11* (5.77%), *BRAF* (3.85%), *PTEN* (9.62%), *MAP2K1* (1.92%), and *FGFR* (1.92%) (Figure 1). Only 14 Patients (26%) carried mutations targetable by available precision medicine therapies (*EGFR* 25 mutations, *BRAF* 2 mutations, and *ALK* 2 mutations), and 25 out of 52 patients presented more than one mutation (Table 1).

### 3.2. Relationship between miRNA Profiles and Mutations in Oncogenes

**Figure 2:**
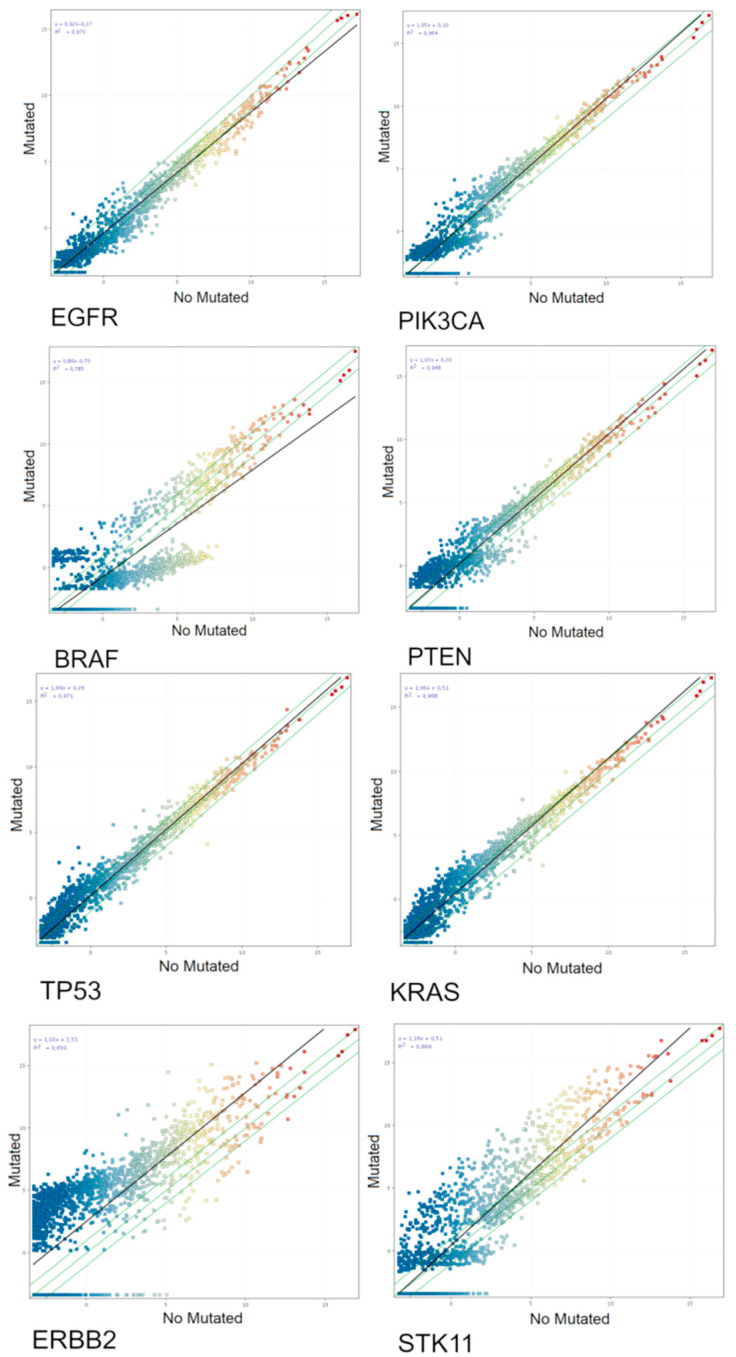
Scatter plot analyses of the miRNA-chip-array results from tumoral lung biopsies of analyzed patients without (horizontal axis) and with (vertical axis) mutations for the oncogenes *TP53*, *KRAS, EGFR, PIK3CA, ERBB2, STK11, BRAF,* and *PTEN*. The miRNA profile was significantly related with the mutational status of the analyzed oncogenes as demonstrated by the miRNA changing their expression more than two-fold falling outside the two-fold variation interval indicated by the diagonal green lines. The slope of the best fit regression line (black diagonal line) indicates the overall trend toward up or downregulation for the mutational status of each oncogene.

From the 38 patients in which both miRNA and mutations were analyzed, 33 were patients carrying at least one mutation. The mutational status affects the miRNA expression. Indeed, the expression of Cancer Related miRNAs was different between mutation-carrier and mutation-free patients, as shown for each mutation by scatter plot analyses (Figure 2).

miRNAs altered in tumoral tissue associated with each oncogene mutation were identified by volcano plot analyses (FC > 2.0, *p* < 0.05). Their identity is reported in the supplementary material (Table S1). The number of cancer-related miRNAs altered in tumoral tissue as associated for each oncogene mutation is reported in Table 2.

### 3.3. Clinical Predictivity of miRNA Profiling

**Figure 3:**
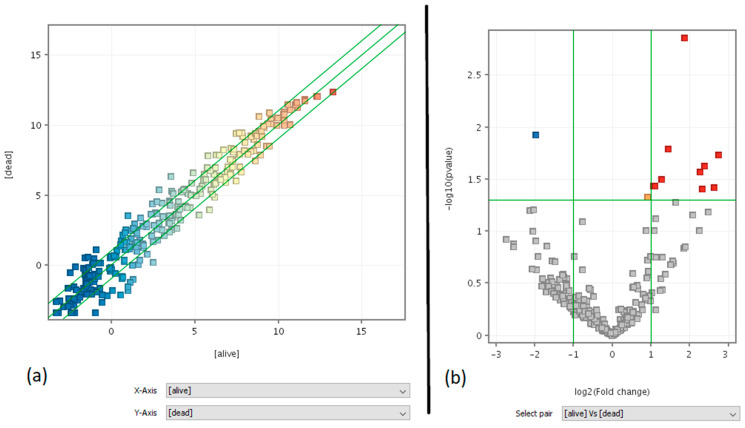
Best-fit line in black, fold change and p-value lines in green. (**a**) Scatter plot analysis = entity list: cancer-related miRNAs (273), interpretation: averaged (alive) vs. (dead), FC ≥ 2.0. (**b**) Moderated T-test volcano plot analysis = entity list: cancer-related miRNAs (273), interpretation: averaged (alive) vs. (dead), without multiple testing correction, *p*-value cut-off = 0.05, fold-change cut-off = 2.0.

In this study: 9 out of 35 monitored patients died within 3 years of the biopsy. We explored whether the miRNA lung tumor expression profile was predictive of the clinical outcome in the following years after surgery. Indeed, the miRNA expression profile in cancer tissue was different between survivors and non-survivors, as shown by the scatter plot (Figure 3a) and volcano plot analyses (Figure 3b). The list of the 11 miRNAs predictive of patient survival (10 up-regulated (red dots) in survivors as compared to non survivors and 1 down-regulated (blue dot)) is reported in Table S2. A prediction model using the list of these 11 miRNAs related to survival and the GeneSpring Neural Network prediction algorithm was run, obtaining an overall accuracy of 0.92 (+0.11), a higher result than those obtained for all miRNA entities (accuracy 0.81). This high accuracy shows the potential of these 11 cancer-related miRNAs from lung biopsies to be used as survival predictors.

**Figure 4:**
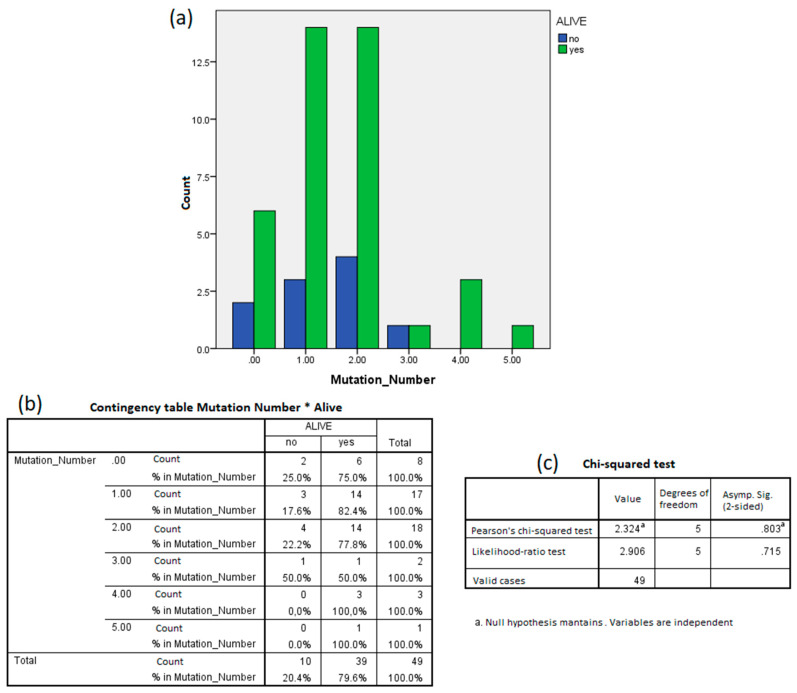
The number of mutations did not predict patient survival. (**a**) Bar plot of the number of patients with zero to five mutations detected separated by survival. (**b**) Same data of (**a**) summarized in a contingency table. (**c**) The Chi-squared test maintained the null hypothesis: the survival and mutation number were independent variables.

Conversely, the mutation status poorly predicted the survival. Indeed, the rate of survivors (47 out of 60) and non-survivors (13 out of 60) was not different between mutation free (10 out of 52) and mutation carrier (42 out of 52) patients. The patient’s detailed information can be found in Table S2. The number of mutations carried by the same patients was not different between survivors and non-survivors as demonstrated by the Chi-squared test (*p* = 0.803) (Figure 4).

### 3.4. Liquid Biopsy: Lung Versus Blood

The intensity of the expression of circulating blood miRNAs from 41 patients was used to classify miRNAs according to their inter-quartile average intensity expression (0–25% = undetectable, 26–50% = low, 51–75% = intermediate, and 76–100% = high). Out of the 273 *Cancer Related miRNAs,* 217 entries were also present in miRNA blood arrays. Venn diagram analysis indicated that the majority (*n* = 121) were undetectable and expressed at a low, 43 were detectable at an intermediate level, and 53 at a high level, i.e., in the upper quartile of the distribution (Table S3).

Accordingly, a signature of 53 cancer related miRNAs present in the blood with a high expression was identified. The panel of these highly detectable miRNAs was compared with their use as possible biomarkers in blood and serum as available in the existing literature (Table 3). Let-7b-5p, miR-150-5p, miR-22-5p, miR-26a-5p, miR-30b-5p, miR-30c-5p, and miR-486-3p were also present in other studies examining circulating miRNAs in the blood as lung cancer biomarkers [15,16,17,18].

A similar approach was used to identify the presence in the plasma of lung miRNAs predictive of clinical outcome (survival). Out of the 11 predictive miRNAs identified in the lung cancer tissue (Table S2), Venn diagram analysis indicated that four were expressed at a high level (high inter-quartile intensity) in the plasma. These miRNAs were miR-23a-5p, miR-147b, miR-371b-5p, and miR-2861.

## 4. Discussion and Conclusions

The identification of novel biomarkers based on miRNA profiles from accessible biological samples, like blood, would help in the near future for a better understanding of a patient’s health state. Outcomes, like better malignant tumor tissue early detection, over time therapy effectiveness prediction, and patient survival prediction rates, may become a reality. The identification of useful circulating miRNAs for predictive outcomes may require models based on standardized tissue-specific and blood-based profiles in oncologic patients.

As seen in our results, the presence of different mutations can modify the scatter plot in each case. From the cancer-related miRNAs significantly altered in each mutation, only four were predicted by TargetScan to target the considered genes as follows: hsa-miR-15b-3p and hsa-miR-21-3p for *KRAS*, hsa-miR-548aa for *STK11*, and hsa-miR-205-3p for *TP53*. This suggests that the dysregulation in these miRNAs may worse the mutation condition.

Indeed, functional miRNAs may silence the expression of mutated oncogenes by destroying their encoded mRNA. Accordingly, whenever the miRNA machinery is well functioning, oncogene mutation does not bear relevance for phenotypic changes and progression of the cell in the carcinogenesis process. Conversely, whenever these miRNAs are altered, their efficacy in neutralizing the mRNAs encoded by mutated oncogenes is lost, and oncogene mutation acquires relevance and efficacy for changing cell phenotypes and moving the carcinogenesis process forward.

We found 11 miRNAs as statistically significant predictors of the patients’ dead within 3 years: hsa-miR-1227-5p, hsa-miR-147b, hsa-miR-187-5p, hsa-miR-23a-5p, hsa-miR-2861, hsa-miR-3663-5p, hsa-miR-371b-5p, hsa-miR-6068, hsa-miR-6075, hsa-miR-6771-5p, and hsa-miR-7704. At the same time, our analysis confirmed that survival was not correlated to the number of oncogene mutations.

From this list, four miRNAs (miR-187-5p, miR-147b, miR-2861, and miR-6075) appear to be the most promising survival markers. Researchers observed that miR-187-5p suppresses cancer cell progression in non-small cell lung cancer (NSCLC) through the down-regulation of *CYP1B1* [19,20], and that miR-147b promotes lung adenocarcinoma cell aggressiveness through glycoprotein 4 (*MFAP4*) regulation [21]; miR-2861 was proposed as a biomarker of lung cancer stem cells [22]; and miR-6075 was used as a biomarker for lung cancer high-accuracy diagnosis prediction models [23]. As our results demonstrated, from the 59-miRNA signature for blood, the best candidates were let-7b-5p, miR-150-5p, miR-22-5p, miR-26a-5p, miR-30b-5p, miR-30c-5p, and miR-486-3p, as they are also present in other studies regarding circulating miRNA biomarkers in the blood for lung cancer.

The silencing role of microRNA on the expression of mutated oncogenes is well established mainly for lung carcinogenesis. The k-ras let-7 interaction is the best typical example. However, the interaction of miRNAs with the target mRNA may be either specific or unspecific according to the number of complimentary nucleotides recognized on targeted sequences. We cannot exclude that, if the mutations of the oncogenes occurred in their coding regions, this situation could generate escaping mutants not recognized by the miRNA. However, thus far, this situation has not yet been demonstrated, at variance with the presence of experimental data clearly indicating the inhibitory role of miRNA toward oncogene expression.

The main finding of this study was that the evaluation of oncogene mutations alone was poorly predictive of the clinical outcome. The predictive potential was remarkably increased when oncogenes mutation were evaluated in parallel with the analysis of related miRNAs. This approach may be used for the personalized screening of lung cancer to identify high risk subjects to undergo early diagnosis screening by spiral TAC. Indeed, for practical and economic reasons, this approach cannot be applied to the whole population but only to high risk subjects identified by predictive biomarkers.

The obtained results may also be useful for lung cancer treatment, which is currently focused only on the oncogene mutational status. Our results provide evidence that oncogene mutation does not per se directly reflect on clinical outcomes and cancer behavior. These variables are determined by other important contributing factors, such as miRNA expression. The comparative analysis of oncogene mutations and miRNA alteration may be useful to identify responders to treatments specifically targeting oncogene mutations or those developing resistance to these treatments.

A remarkable results of our study is the correspondence of the results between the liquid biopsy analysis and corresponding data for the target tissue, i.e., the lung. Indeed, we evaluated in parallel in the same patient both blood and lung tissues. The cancer-related signature was evaluated by comparing the miRNA expression between the cancer and healthy surrounding tissue. This signature accounted for 273 miRNAs, 53 of them being well detectable (i.e., in the highest quartile of the distribution) and also by liquid biopsy in the blood plasma. Among these 53 miRNAs, 7 were further confirmed as lung cancer biomarkers in the blood by other independent studies. These results represents a step forward to identify a miRNA blood signature applicable to the early diagnosis of lung cancer.

Overall, the herein reported results provide evidence that the parallel analyses of miRNA and oncogene mutations was more predictive of lung cancer occurrence that the single analysis of only one of these two biomarkers. A tight interconnection between the pattern of miRNA alteration and the mutations occurring was detected, a finding demonstrating the interplay between genetic damage and the postgenomic control exerted by the miRNA machinery. The predictivity of the clinical outcome (survival) was good for the postgenomic miRNA analysis and undetectable for the oncogene mutation analysis. This analysis could be executed by the non-invasive sampling of blood plasma given the fact that both oncogene mutations and specific lung cancer miRNAs can be detected in this body fluid. Such an approach could represent a new tool applicable to cancer preventive and predictive medicine.

These findings support the use of parallel miRNA and oncogene mutation analysis as a new tool to provide clinical and preventive interventions tailored for the individual situation of each subject or patients, thus, realizing a practical approach of personalized medicine applicable to cancer prevention.

## Figures and Tables

**Table 1:** Frequency of single, double, triple, quadruple, and quintuple mutations in lung biopsies from the analyzed patients.

Number of Mutations	Frequency	Percentage
0	10	19.2
1	17	32.7
2	19	36.5
3	2	3.8
4	3	5.8
5	1	1.9
Total	52	100.0

**Table 2:** A number of miRNAs significantly changed their expression according to the mutational status of each oncogene. This table lists the number of these miRNAs in patients carrying oncogene mutations.

Gene	Mutated/Total Patients	Number of Entities (Altered miRNAs)	Up	Down	miRNAs Targeting Mutated Oncogene
*BRAF*	2/33	7	1	6	0
*EGFR*	9/33	1	0	1	0
*ERBB2*	1/33	-	-	-	-
*ERBB4*	1/33	-	-	-	-
*FGFR3*	1/33	-	-	-	-
*KRAS*	10/33	13	13	0	2 (hsa-miR-15b-3p, hsa-miR-21-3p)
*NOTCH1*	3/33	1	1	0	0
*PIK3CA*	3/33	0	0	0	0
*PTEN*	2/33	0	0	0	0
*STK11*	2/33	31	30	1	1 (hsa-miR-548aa)
*TP53*	10/33	4	4	0	1 (hsa-miR-205-3p)

**Table 3:** Cancer-related miRNAs present in the blood with a high expression falling in the upper quartile of the distribution of intensity expression. The intensity of the miRNA expression was reported in fluorescence units as detected by microarray analysis (middle column). The miRNAs found in our study were compared with the bibliography to evaluate if their dysregulation in the blood could be considered as potential markers for lung cancer (right column).

Tumoral miRNAs in Blood	Average Signal in Blood Plasma Microarray (Fluorescence Units)	Reference in Literature
hsa-let-7b-5p	1556.03	[16,17]
hsa-let-7e-5p	2570.68	[15]
hsa-let-7g-5p	1591.89	ND
hsa-miR-103a-3p	1966.77	ND
hsa-miR-107	2307.96	ND
hsa-miR-1247-5p	1619.35	ND
hsa-miR-143-3p	1517.48	ND
hsa-miR-147b	2867.22	ND
hsa-miR-150-5p	1728.39	[15]
hsa-miR-151a-5p	2375.45	ND
hsa-miR-151b	2375.45	ND
hsa-miR-181a-2-3p	1714.68	ND
hsa-miR-183-3p	4515.51	ND
hsa-miR-184	3097.26	ND
hsa-miR-185-5p	2809.76	ND
hsa-miR-193a-5p	1482.98	ND
hsa-miR-22-5p	1889.39	[15,17]
hsa-miR-224-3p	1805.07	ND
hsa-miR-23a-5p	2880.99	ND
hsa-miR-26a-5p	1497.29	[17]
hsa-miR-2861	3086.05	ND
hsa-miR-29b-2-5p	1481.13	ND
hsa-miR-30b-5p	2117.35	[16]
hsa-miR-30c-2-3p	2919.97	ND
hsa-miR-30c-5p	3299.46	[17,18]
hsa-miR-3149	2367.91	ND
hsa-miR-361-3p	1454.65	ND
hsa-miR-371b-5p	12,583.51	ND
hsa-miR-424-5p	1660.83	ND
hsa-miR-4252	1558.12	ND
hsa-miR-4290	1875.54	ND
hsa-miR-4306	7145.12	ND
hsa-miR-4324	1530.87	ND
hsa-miR-4440	1537.86	ND
hsa-miR-4443	3859.37	ND
hsa-miR-4481	1605.44	ND
hsa-miR-450a-5p	4646.58	ND
hsa-miR-4516	11,603.31	ND
hsa-miR-452-5p	1629.61	ND
hsa-miR-4532	13,231.17	ND
hsa-miR-4634	1884.47	ND
hsa-miR-483-3p	3177.46	ND
hsa-miR-486-3p	2337.52	[14,18]
hsa-miR-490-3p	1882.31	ND
hsa-miR-505-5p	3775.67	ND
hsa-miR-516b-5p	5121.31	ND
hsa-miR-548aa	3480.4	ND
hsa-miR-548q	1453.76	ND
hsa-miR-642b-5p	2070.84	ND
hsa-miR-664b-3p	1539.91	ND
hsa-miR-744-5p	3112.18	ND
hsa-miR-99a-3p	1503.5	ND
hsa-miR-99b-5p	1575.63	ND

ND, Not Detected.

## Data Availability

The datasets used and/or analysed during the current study are available from the corresponding author on reasonable request.

## Supplementary Materials

The following are available online at https://www.mdpi.com/2075-4426/11/3/182/s1, Table S1: Cancer Related miRNAs altered (FC ≥ 2, *p* ≤ 0.05) in Volcano Plot Analysis between average signal in samples with non-small cell lung cancer vs. small cell lung cancer, Table S2: Cancer Related miRNAs run on a Moderated T-Test Volcano Plot analysis (FC ≥ 2, *p* ≤ 0.05) for each environmental exposure: (a) passive smoke at home (No vs. Yes), (b) passive smoke at work (No vs. Yes); (c) airborne car traffic pollution (low vs. high); (d) volcano ashes (>60 Km vs. ≤60 Km); (e) radon risk (according to house type low vs. high), Table S3: Cancer Related miRNAs altered (FC ≥ 2, *p* ≤ 0.05) in Volcano Plot Analysis between average signal in samples of patients alive vs. dead within 3 years since biopsy.
