# Supplementary material for: Relationship between the miRNA Profiles and Oncogene Mutations in Non-Smoker Lung Cancer. Relevance for Lung Cancer Personalized Screenings and Treatments
Source: J Pers Med. 2021 Mar 5;11(3):182. doi: 10.3390/jpm11030182 (PMC7999775; doi:10.3390/jpm11030182)
Supplement: Supplementary file 1 [file jpm-11-00182-s001.pdf]

Table S1. ***Cancer Related miRNAs*** altered (FC>= 2, p<=0.05) in Volcano Plot Analysis between average signal in samples with non-small cell lung cancer vs. small cell lung cancer.

systematic_name	p-value	Regulation	FC
hsa-miR-1238-5p	0.011302715	up	9.62
hsa-miR-1296-5p	0.007524842	up	8.62
hsa-miR-1306-3p	0.021063296	up	8.58
hsa-miR-205-3p	4.19297E-06	up	220.64
hsa-miR-2277-3p	0.00932873	up	10.59
hsa-miR-3149	0.000521124	up	25.38
hsa-miR-326	0.019960763	down	-8.85
hsa-miR-4290	0.000451764	up	16.39
hsa-miR-4440	0.002965666	up	10.05
hsa-miR-4443	0.03872927	up	2.41
hsa-miR-4481	0.015899722	up	7.33
hsa-miR-4716-5p	0.000232035	up	20.00
hsa-miR-4763-5p	0.008582705	up	8.26
hsa-miR-4793-3p	0.002927522	up	16.70
hsa-miR-483-3p	0.002941658	up	13.22
hsa-miR-504-3p	0.002079689	up	14.95
hsa-miR-595	0.000426941	up	21.36
hsa-miR-6730-3p	0.016724579	up	6.91
hsa-miR-6743-3p	0.043577574	up	7.71
hsa-miR-6779-3p	0.016434822	up	8.81
hsa-miR-6794-3p	0.005262722	up	7.19
hsa-miR-6817-5p	0.044506542	up	7.97
hsa-miR-6826-5p	0.02230857	up	2.63
hsa-miR-6886-3p	0.014682693	up	12.86
hsa-miR-6891-3p	0.01124693	up	7.93
hsa-miR-7108-3p	0.012961295	up	6.21

Table S2. ***Cancer Related miRNAs*** run on a Moderated T-Test Volcano Plot analysis (FC>= 2, p<=0.05) for each environmental exposure: (a) passive smoke at home (No vs. Yes), (b) passive smoke at work (No vs. Yes); (c) airborne car traffic pollution (low vs. high); (d) volcano ashes (>60Km vs. <=60Km); (e) radon risk (according to house type low vs. high).

systematic_name	p-value	Regulation ([low] Vs [high])	FC ([low] Vs [high])	environmental exposure
hsa-miR-2277-3p	0.01829	down	-7.38783	passive_smoking_at_home
hsa-miR-328-3p	0.013113	down	-5.98362	passive_smoking_at_home
hsa-miR-4254	0.002496	down	-14.3598	passive_smoking_at_home
hsa-miR-483-3p	0.015154	down	-7.43976	passive_smoking_at_home
hsa-miR-491-3p	0.003077	down	-11.924	passive_smoking_at_home
hsa-miR-6743-3p	0.022234	down	-9.42842	passive_smoking_at_home
hsa-miR-6779-3p	0.009505	down	-9.89658	passive_smoking_at_home

hsa-miR-6886-3p	0.008845	down	-13.8967	passive_smoking_at_home
hsa-miR-4695-3p	0.04655064	down	-5.80325	Passive_smoking_at_work
hsa-let-7a-5p	0.041112	up	7.4232	Vehicle_traffic_at_home
hsa-let-7b-5p	0.011125	up	4.773517	Vehicle_traffic_at_home
hsa-let-7c-5p	0.015205	up	5.49175	Vehicle_traffic_at_home
hsa-miR-100-5p	0.040598	up	7.583029	Vehicle_traffic_at_home
hsa-miR-103a-3p	0.039793	up	7.255231	Vehicle_traffic_at_home
hsa-miR-125a-5p	0.034176	up	5.446658	Vehicle_traffic_at_home
hsa-miR-125b-5p	0.021168	up	6.086423	Vehicle_traffic_at_home
hsa-miR-130a-3p	0.048873	up	6.662414	Vehicle_traffic_at_home
hsa-miR-133b	0.047599	up	9.969255	Vehicle_traffic_at_home
hsa-miR-142-3p	0.046327	up	8.855112	Vehicle_traffic_at_home
hsa-miR-146a-5p	0.023557	up	8.37029	Vehicle_traffic_at_home
hsa-miR-150-5p	0.039885	up	10.98931	Vehicle_traffic_at_home
hsa-miR-151b	0.015652	up	8.787968	Vehicle_traffic_at_home
hsa-miR-15b-5p	0.03082	up	8.508455	Vehicle_traffic_at_home
hsa-miR-16-2-3p	0.044513	up	5.532554	Vehicle_traffic_at_home
hsa-miR-185-5p	0.039332	up	5.07439	Vehicle_traffic_at_home
hsa-miR-199a-5p	0.04242	up	4.570111	Vehicle_traffic_at_home
hsa-miR-203a-3p	0.02749	up	18.03255	Vehicle_traffic_at_home
hsa-miR-204-5p	0.041068	up	9.511877	Vehicle_traffic_at_home
hsa-miR-20a-5p	0.045853	up	8.663452	Vehicle_traffic_at_home
hsa-miR-214-3p	0.026044	up	5.304047	Vehicle_traffic_at_home
hsa-miR-221-3p	0.017622	up	8.297637	Vehicle_traffic_at_home
hsa-miR-221-5p	0.013803	up	17.72528	Vehicle_traffic_at_home
hsa-miR-222-3p	0.013626	up	3.554951	Vehicle_traffic_at_home
hsa-miR-223-3p	0.012787	up	15.80766	Vehicle_traffic_at_home
hsa-miR-224-3p	0.017806	up	9.024755	Vehicle_traffic_at_home
hsa-miR-23a-3p	0.035768	up	4.962604	Vehicle_traffic_at_home
hsa-miR-23a-5p	0.011235	up	8.435381	Vehicle_traffic_at_home
hsa-miR-30c-2-3p	0.004407	up	5.743132	Vehicle_traffic_at_home
hsa-miR-3188	0.044807	up	3.079721	Vehicle_traffic_at_home
hsa-miR-324-3p	0.035711	up	2.143047	Vehicle_traffic_at_home
hsa-miR-339-5p	0.008492	up	7.478019	Vehicle_traffic_at_home
hsa-miR-342-3p	0.018368	up	6.661599	Vehicle_traffic_at_home
hsa-miR-365a-3p	0.036355	up	6.536557	Vehicle_traffic_at_home
hsa-miR-4306	0.002998	up	3.347996	Vehicle_traffic_at_home
hsa-miR-4324	0.033328	up	5.706607	Vehicle_traffic_at_home
hsa-miR-4516	0.006668	up	2.859822	Vehicle_traffic_at_home
hsa-miR-452-5p	0.014409	up	7.888119	Vehicle_traffic_at_home
hsa-miR-4532	0.014752	up	2.258895	Vehicle_traffic_at_home
hsa-miR-455-3p	0.009444	up	15.94311	Vehicle_traffic_at_home
hsa-miR-4634	0.030527	up	2.128231	Vehicle_traffic_at_home
hsa-miR-4655-3p	0.021817	up	6.810059	Vehicle_traffic_at_home
hsa-miR-4731-3p	0.01469	up	6.153936	Vehicle_traffic_at_home
hsa-miR-501-5p	0.012	up	9.779916	Vehicle_traffic_at_home
hsa-miR-505-5p	0.013655	up	7.634665	Vehicle_traffic_at_home
hsa-miR-517a-3p	0.027523	up	5.597306	Vehicle_traffic_at_home
hsa-miR-517c-3p	0.025637	up	5.422509	Vehicle_traffic_at_home
hsa-miR-582-5p	0.044966	up	13.7203	Vehicle_traffic_at_home
hsa-miR-6895-5p	0.006121	up	7.657433	Vehicle_traffic_at_home

hsa-miR-744-5p	0.019918	up	10.30978	Vehicle_traffic_at_home
hsa-miR-98-5p	0.035529	up	13.94902	Vehicle_traffic_at_home
hsa-miR-99b-5p	0.040104	up	4.172732	Vehicle_traffic_at_home
hur_6	-	-	-	Vehicle_traffic_at_home
hsa-miR-133a-3p	0.017678	up	5.314951	km_from_etna_50_median
hsa-miR-135a-5p	0.034151	up	5.869018	km_from_etna_50_median
hsa-miR-193a-5p	0.044354	up	2.526421	km_from_etna_50_median
hsa-miR-27a-5p	0.023292	down	-3.61896	km_from_etna_50_median
hsa-miR-29b-2-5p	0.014229	up	4.74146	km_from_etna_50_median
hsa-miR-340-5p	0.033516	up	6.845372	km_from_etna_50_median
hsa-miR-3607-3p	0.012756	up	5.837188	km_from_etna_50_median
hsa-miR-361-3p	0.025633	up	4.868399	km_from_etna_50_median
hsa-miR-449a	0.026818	up	10.2289	km_from_etna_50_median
hsa-miR-4770	0.03923	up	4.277719	km_from_etna_50_median
hsa-miR-500a-3p	0.037476	up	4.086222	km_from_etna_50_median
hsa-miR-500b-5p	0.008137	up	3.92188	km_from_etna_50_median
hsa-miR-505-5p	0.030313	up	4.50934	km_from_etna_50_median
hsa-miR-5701	0.019156	up	6.29237	km_from_etna_50_median
hsa-miR-628-5p	0.022147	up	5.505154	km_from_etna_50_median
hsa-miR-642b-5p	0.016176	up	2.964167	km_from_etna_50_median
hsa-miR-6516-3p	0.041004	up	5.372472	km_from_etna_50_median
hsa-miR-652-3p	0.027532	up	4.759585	km_from_etna_50_median
hsa-miR-664a-3p	0.042052	up	2.812089	km_from_etna_50_median
hsa-miR-664b-3p	0.023029	up	3.220316	km_from_etna_50_median
hsa-miR-874-5p	0.003118	up	5.434077	km_from_etna_50_median
hsa-miR-16-2-3p	0.024773	down	-9.45544	Radon_risk_ Home_type
hsa-miR-182-3p	0.033553	down	-7.05349	Radon_risk_ Home_type
hsa-miR-22-5p	0.030786	down	-15.112	Radon_risk_ Home_type
hsa-miR-221-5p	0.016387	down	-23.7398	Radon_risk_ Home_type
hsa-miR-30c-2-3p	0.012436	down	-6.19418	Radon_risk_ Home_type
hsa-miR-3660	0.001222	down	-29.6997	Radon_risk_ Home_type
hsa-miR-4306	0.005655	down	-3.77123	Radon_risk_ Home_type
hsa-miR-4440	0.033708	down	-7.56958	Radon_risk_ Home_type
hsa-miR-4443	0.0132	down	-3.02148	Radon_risk_ Home_type
hsa-miR-452-5p	0.030769	down	-8.7803	Radon_risk_ Home_type
hsa-miR-454-3p	0.01435	down	-33.4518	Radon_risk_ Home_type
hsa-miR-455-3p	0.038657	down	-14.6433	Radon_risk_ Home_type
hsa-miR-4793-3p	0.002683	down	-13.7501	Radon_risk_ Home_type
hsa-miR-598-3p	0.025903	down	-15.7451	Radon_risk_ Home_type
hsa-miR-6500-5p	0.035551	down	-6.86215	Radon_risk_ Home_type
hsa-miR-6826-5p	0.037038	down	-2.57302	Radon_risk_ Home_type
hsa-miR-6872-3p	0.020769	down	-3.46987	Radon_risk_ Home_type
hsa-miR-7159-5p	0.031503	down	-5.91743	Radon_risk_ Home_type
hsa-miR-98-5p	0.020851	down	-28.8801	Radon_risk_ Home_type

Table S3. ***Cancer Related miRNAs*** altered (FC>= 2, p<=0.05) in Volcano Plot Analysis between average signal in samples of patients alive vs. dead within 3 years since biopsy.

**systematic_name**	**p-value**	**Regulation**	**FC**

hsa-miR-1227-5p	0.03180667	up	2.42
hsa-miR-147b	0.011820709	down	-3.96
hsa-miR-187-5p	0.03807269	up	6.18
hsa-miR-23a-5p	0.01835003	up	6.71
hsa-miR-2861	0.036854673	up	2.08
hsa-miR-3663-5p	0.03956902	up	5.03
hsa-miR-371b-5p	0.001374278	up	3.66
hsa-miR-6068	0.016317874	up	2.72
hsa-miR-6075	0.02674605	up	4.83
hsa-miR-6771-5p	0.023678219	up	5.23
hsa-miR-7704	0.03648895	up	2.15
